# Variation in exposure to *Anopheles gambiae* salivary gland peptide (gSG6-P1) across different malaria transmission settings in the western Kenya highlands

**DOI:** 10.1186/1475-2875-11-318

**Published:** 2012-09-10

**Authors:** Kingsley Badu, Joram Siangla, John Larbi, Bernard W Lawson, Yaw Afrane, John Ong’echa, Franck Remoue, Guofa Zhou, Andrew K Githeko, Guiyun Yan

**Affiliations:** 1 Department of Theoretical and Applied Biology, College of Sciences, Kwame Nkrumah, University of Science & Technology, Kumasi, Ghana; 2 Center for Global Health Research, Kenya Medical Research Institute, Kisumu, Kenya; 3 Walter Reed Project, United States Army Medical Research Unit-Kenya, Kisumu; 4 Institute of Research for Development (IRD), MIVEGEC unit - (IRD224-UM1-UM2-CNRS 5290), Cotonou, Benin; 5 Program in Public Health, College of Health Sciences, University of California at Irvine, Irvine, CA, 92697, USA

## Abstract

**Background:**

The existing metrics of malaria transmission are limited in sensitivity under low transmission intensity. Robust surveillance systems are needed as interventions to monitor reduced transmission and prevention of rapid reintroduction. Serological tools based on antibody responses to parasite and vector antigens are potential tools for transmission measurements. The current study sought to evaluate antibody responses to *Anopheles gambiae* salivary gland peptide (gSG6- P1), as a biomarker of human exposure to *Anopheles* bites, in different transmission settings and seasons. The comparison between anti-MSP-1_19_ IgG immune responders and non-responders allowed exploring the robustness of the gSG6-P1 peptide as a surveillance tool in an area of decreasing malaria transmission.

**Methods:**

Total IgG levels to gSG6-P1 were measured in an age-stratified cohort (< 5, 5–14 and ≥ 15 years) in a total of 1,366 participants from three localities in western Kenya [Kisii (hypoendemic), Kakamega (mesoendemic), and Kombewa (hyperendemic)] including 607 sera that were additionally tested for MSP-1_19_ specific responses during a low and a high malaria transmission seasons. Antibody prevalence and levels were compared between localities with different transmission intensities. Regression analysis was performed to examine the association between gSG6-P1 and MSP-1_19_ seroprevalence and parasite prevalence.

**Result:**

Seroprevalence of gSG6-P1 in the uphill population was 36% while it was 50% valley bottom (χ^2^ = 13.2, df = 1, p < 0.001). Median gSG6-P1 antibody levels in the Valley bottom were twice as high as that observed in the uphill population [4.50 vs. 2.05, p < 0.001] and showed seasonal variation. The odds of gSG6-P1 seropositives having MSP-1_19_ antibodies were almost three times higher than the odds of seronegatives (OR = 2.87, 95% CI [1.977, 4.176]). The observed parasite prevalence for Kisii, Kakamega and Kombewa were 4%, 19.7% and 44.6% whilst the equivalent gSG6-P1 seroprevalence were 28%, 34% and 54%, respectively.

**Conclusion:**

The seroprevalence of IgG to gSG6-P1 was sensitive and robust in distinguishing between hypo, meso and hyper transmission settings and seasonal fluctuations.

## Background

Accumulating evidence indicate that malaria burden in Africa is declining [1,2]. Several countries that previously had high malaria burden have seen over 50% reduction in malaria burden within the past ten years, including Eritrea, Rwanda, Zanzibar [3], Pemba [4], Tanzania mainland [5], Kenya [6], Gambia [7], Zambia [8], and Swaziland [9]. Three countries, including Morocco, in Africa were certified as malaria-free in 2011 [10]. Moreover, a longitudinal decline in the density of malaria vectors was observed during an 11-year study period, in spite of the absence of organized vector control [11]. Guerra and others have estimated that there are about 1 billion people currently living under unstable or extremely low malaria risk globally. These areas are amenable for malaria elimination [12]. As programmes successfully reduce transmission to near elimination levels, the measurement of malaria-associated morbidity and mortality as a means of tracking reducing burden will become difficult and insensitive. Novel approaches to surveillance are, therefore, necessary to ensure that once elimination has been achieved, it is not threatened by a rapid reintroduction [13]. People living in areas of unstable or extremely low malaria risk may lose the ability of maintaining naturally acquired immunity [14]. This presents a special challenge, i.e., the risk of possible catastrophic rebound such as the one occurred in the highlands of Madagascar in the 1980s where an epidemic killed more than 40,000 people [15]. Thus, the quest for sensitive and robust surveillance tools has become imperative. Such surveillance tools are needed as an intervention to reduce transmission, to measure transmission interruption and maintenance of zero transmission; the tools should also be useful in mapping the risk of focal residues of transmission to enable targeted control. Unfortunately, the existing metrics of malaria transmission have serious limitations when transmission is approaching zero. The entomological inoculation rate (EIR), the gold standard of malaria transmission intensity (MTI) [16], becomes difficult, expensive, and sometimes virtually impossible to measure when transmission is very low [17,18].

Serological tools based on antibody responses to parasite and vector antigens are potentially valuable for robust transmission measurement [19-21]. Particularly, Merozoite Surface Protein 1 (MSP 1_19_) seroconversion rates have been shown to correlate with malaria transmission intensity (EIR) [22,23]. MSP-1_19_ seroprevalence and antibody level is robust and sensitive in distinguishing malaria exposures at different altitudes, age groups, and proximity to mosquito breeding habitats in populations separated by only 5 km apart [24]. The parallel measure of the antibody response to *Anopheles* salivary antigen would be especially convenient, because it will allow for assessment of *Anopheles* exposure in children, which is ethically unfeasible by human landing catches. Moreover, serological markers of exposure to *Anopheles* bites would represent a complementary tool in low malaria transmission areas for the monitoring of control interventions based on anti-vector measures [17,25]. The IgG response to whole saliva extracts of *Anopheles gambiae* has been observed as a marker of exposure to *An. gambiae* bite, and high anti-saliva IgG levels is a predictive indicator of malaria morbidity [26,27]. The *An. gambiae* salivary gland gSG6 protein and derived P1 peptide are specific to *An. gambiae* and elicit specific antibody response in the human host [28,29]. It is antigenic in travelers transiently exposed to *Anopheles* bites in malaria endemic areas of Africa [30]. The gSG6 protein has been recently reported to have the potential to represent a general epidemiological marker of exposure since it shares 99% and 80% identity with *Anopheles arabiensis* and *Anopheles funestus,* respectively, which constitutes the main Afro-tropical malaria vectorial system [29,31]. The synthetic peptide (gSG6-P1) derived from *An. gambiae* salivary recombinant protein gSG6-P1 is reportedly highly specific to *Anopheles* species and immunogenic [21,29] and its synthetic nature guarantees high reproducibility for the assay [21]. It is also a biomarker in low exposure area [28] and specific to *An. funestus* bites [32]. There is as yet, lack of information on the value of gSG6-P1 as a surveillance tool in assessing the risk of exposure to malaria parasites at the population and individual level. This study thus sought to evaluate anti-gSG6- P1 IgG responses in *Pf*MSP-1_19_ responders and non-responders across different altitudes under high, moderate and low transmission settings across different age groups and seasons. The robustness of the gSG6-P1 as a biomarker of parasite exposure and the possibility of utilizing the biomarker as a surveillance tool in an era of decreasing malaria transmission where traditional tools become insensitive and unfeasible to track malaria transmission is reported.

## Methods

### Study site

**Figure 1:**
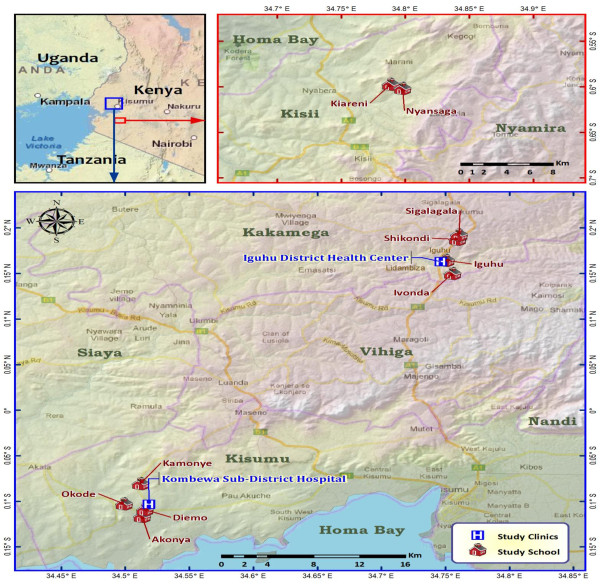
Shows the Map of the study sites.

The study was conducted in three sentinel sites in western Kenya, comprising hyperendemic site of Kombewa in Kisumu County, mesoendemic site of Iguhu in the Kakamega County, and hypoendemic site Marani village in the Kisii County. Iguhu and Kisii are sites in the highlands and Kombewa is in a lowland area of the Lake Victoria basin, Figure 1.

Iguhu area (34°45’9” E, 00°10’9” N, 1,430–1,580 m above sea level) was subdivided into two locations: an uphill site and valley bottom site, these have been described to have distinct malaria epidemiology [24,33,34]. The area is characterized by mosaic land use types: the hill is mostly maize farms dotted by patches of tea plantation. A slow flowing Yala River runs through the flood prone flat valley bisecting the community and because several swamps are located along the Yala River, mosquito larval habitats are common at the river banks near the valley bottom. The entomological inoculation rate (EIR) at the valley was 16.6 during 2003–2004 [35], while it was 0.04 at in the uphill area during 1999–2000 [33]. Marani (34° 48’9” E, 00°35’9” S, 1,540–1,740 m a.s.l) in the Kisii county, is located on the highland plateau adjacent to the Lake Victoria Basin. Steep, gently sloping hills and undulating topography characterizes the area. The highland area has small patches of forests along the rivers and streams, which are remnants of a larger forest that has been cleared for cultivation and pasture. The valley is characterized by an efficient drainage system and floods are not common. Marani area is under low and unstable malaria transmission and thus has been described as hypo endemic for malaria [36]. The mean annual rainfall in the western Kenya highlands has been 1800–2000 mm and the mean annual temperature has ranged from 17 to 20°C [37] and the predominant malaria vector species is *An. gambiae* s.s., with insignificant proportion of *An. arabiensis* and *An. funestus*[35]. Other details of the area have been described elsewhere [34]. Kombewa (34°45’ E 0°10’ S, elevation 1,150–1,250 m a.s.l) is in the Kisumu County and lies in the vicinity of the Lake Victoria basin. Kombewa is characterized by a rolling terrain bisected by small streams with poor drainage. It is found within the semi-arid lowland and is warmer and drier compared to the two highland areas. Mean monthly rainfall was 120.7 mm, mean monthly maximum and minimum temperatures were 29.1°C and 18.4°C, respectively. Here malaria is hyper-endemic with *P. falciparum* accounting for more than 95% of infections. *Anopheles gambiae* s.s. and *An. funestus* are the major vectors with recent increased population in *An*. *arabiensis*[36]. The EIR in recent times has been estimated to be of 31.1 infectious bites per person per year [35].

### Parasitological and immunological survey

Two cross-sectional surveys were conducted among age stratified cohorts (≤5, 5–14, ≥15 years) at the Kakamega site during the dry season (February - March) and the rainy season (June-July) in 2009; this survey has been fully described in a previous study [24]. Another survey was conducted among randomly selected school-age children (5–13 years) in all study sites from February to April, 2011. Standard finger prick blood was collected into microvettes (*SARSTEDT* Numbrecht, Germany**)** containing EDTA. This was later spun and about 50μL of plasma was aspirated and stored in -80°C until use. Giemsa-stained blood smears were also made for the estimation of parasite prevalence.

### Study population and demography

A total of 744 sera (322 belonged to the uphill community whilst 422 were from the valley bottom area) available from previous study [24], were successfully tested for IgG response to gSG6-P1; out of this number, 607 of them had known specific antibody responses to MSP-1_19_. Participants were categorized into three age groups, i.e. <5 (n = 49), 5–14 (n = 153) and ≥ 15 years (n = 120); respectively, from the uphill community. Similarly from the valley bottom population, 79, 159 and 189 belonging to the respective age categories <5, 5–14 and ≥ 15 years were tested. Seasonally a total of 384 were sampled in the dry season whilst 360 tested were from the rainy season. The second survey sampled 622 primary school children ages between five and 16, among them 202 participants were from Kombewa, 203 from Iguhu, and 217 from Marani.

### Salivary peptide gSG6-P1

The gSG6-P1 peptide was designed using bioinformatics to maximize its *Anopheles* specificity and its immunogenicity, as previously described [21]. It was synthesized and purified (>95 %) by Genepep SA (St-Jean de Vedas, France) and shipped to Kenya in lyophilized form. Peptides were constituted in 0.23 mL ultra-filtered laboratory grade water and kept frozen at −80°C until use.

### Measurement of anti-human IgG antibodies for gSG6-P1 antigen

Total IgG antibody against the gSG6-P1 antigen was measured by enzyme-linked immunosorbent assay (ELISA) technique described previously [28] with modifications. Briefly, 96-well micro-assay plates (Maxisorp, Roskilde, Denmark) were coated with gSG6-P1 antigen at a concentration of 20 μg/mL and incubated at 37°C for two and half hours. Plates were blocked with 0.5% Casein containing 0.05% Tween20 for 1 hour at 22°C. Test sera, diluted 1:20 in blocking buffer, were added and incubated at 4°C over night. Anti-gSG6- P1 IgG were detected with a horse radish peroxidase (HRP)-conjugate of goat anti-human IgG antibody (Nordic Immunology, Tilburg, Netherlands) diluted 1:10,000 in PBS for1 hour incubation at 22°C. A peroxidase substrate, 2, 2’-azino-bis (3-ethylbenzothiazoline-6-sulphonic acid) or ABTS (Kirkegaard & Perry Laboratories Inc., Gaithersburg, MD) was added and plates incubated for 50 minutes at 22°C. Enzymatic reaction was stopped with 10 μl of 20% SDS (Sigma, St. Louis, MO). Optical density (OD) measurements were taken at 405 nm on a spectrophotometer (*SpectraMAX*^340^PC, Molecular Devices Corporation, USA). Each test sample was assessed in duplicate wells and, in parallel, with a blank well containing no antigen (ODn) to control for non-specific reactions in the plasma and the reagents. IgG levels were expressed as final OD calculated for each serum as the mean OD value with antigen minus the OD value without antigen (ODn). Intra- and inter-assay variation of control samples was below 25%. Sera whose duplicates showed a coefficient of variation (CV) 25% and above were not included in the analysis. The mean OD of unexposed controls (from the USA, N = 30) plus 3 SD was used as cut-off value for seropositivity. The cut off value being 0.35.

### Measurement of anti-human IgG antibodies for *Pf*MSP1_19_ (FVO) antigen

Total IgG antibody to *Pf*MSP1 was measured by indirect ELISA as previously described in our earlier studies [24]. The expression and purification of the *Pf*MSP1 FVO recombinant protein has also been described [38,39]. In brief, 96-well micro-assay plates (Maxisorp, Roskilde, Denmark) were coated with 0.2 μg *Pf*MSP1 FVO antigen (diluted in phosphate buffered saline) and incubated overnight at 4°C. After blocking, test sera were added in triplicate wells and serially diluted from 1:50 to 1:64,000. Plates were incubated for 2 h at 22°C and HRP conjugate of goat antihuman IgG (KPL, Gaithersburg, MD) added. After 1 h incubation, ABTS (Kirkegaard & Perry Laboratories Inc., Gaithersburg, MD) was added and incubated for 1 h at 22°C. The enzymatic reaction was stopped by adding 10μL of 20% SDS (Sigma, St. Louis, MO). Plates were washed and the OD measurements were taken at 414 nm on a spectrophotometer (*SpectraMAX*^340^PC, Molecular Devices Corporation, USA). Serial dilutions were used to fit a four-parameter curve using SoftMax Pro v5.3 (Molecular Devices). Results were expressed in titer values, the titer endpoint being defined in this study as the calculated serum dilution yielding an OD of 1.0.

### Data analysis

Seroprevalence was defined as the number of positive responders to a specific antigen out of the total number tested. Differences in the proportion of seroprevalence of gSG6-P1 between age-stratified, uphill and valley residents were compared by the χ^2^ test with p < 0.05 considered statistically significant. The Mann–Whitney test was used to test if medians of antibody levels were different between localities. Multinomial logistic regression was used to examine the association between gSG6-P1 and MSP-1_19_ seroprevalence adjusting for age in the population. Linear regression was used to examine the trend of parasite prevalence and age at different localities ODs of gSG6-P1.All data were analysed and graphed using GraphPad Prism software (San Diego, CA, USA).

### Scientific and ethical considerations

Scientific and ethical clearance was granted by the scientific and ethical review committee of Kenya Medical Research Institute and University of California at Irvine. Participants were enrolled from the primary schools in the study sites through the primary school administrators with the permission of the division office of the Ministry of Health. Participants were recruited into the study after their parents/guardians gave informed consent. Prospective participants were excluded from the study if they were unwilling to participate; asymptomatic infections were not treated with anti-malarial regimen in line with the standard malaria treatment guidelines from the Ministry of Health of Kenya, symptomatic participants were referred to the local government hospitals or clinics for diagnosis and treatment free of charge.

## Results

### Specific IgG responses to gSG6-P1 in valley and uphill communities and its association to MSP-1_19_ seroprevalence

**Figure 2:**
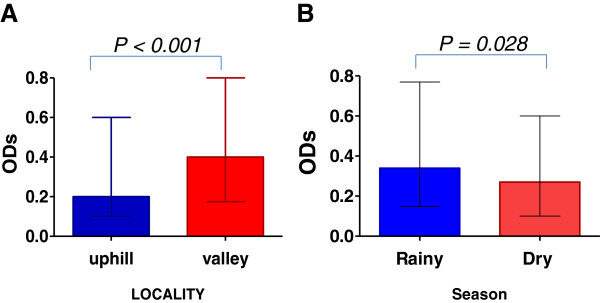
**Shows Bar graphs of specific Median humoral responses to gSG6-P1 in different localities and seasons.A**: responses between Uphill (n = 322) and valley (n = 422) residents. Mann Whitney: P = 0.0002. **B**: responses between and Rainy (n = 360) Dry (n = 384) seasons. Mann Whitney: P = 0.028. Error Bars show inter-quartile ranges (Lower 25 %- Upper 75 %).

There were marked differences in seroprevalence as well as IgG levels to gSG6-P1 peptide between the uphill and valley populations which were statistically significant. The overall seroprevalence in the uphill population was 36% and that of in the valley population was 50% (χ^2^ = 13.2 p <0.001). Median antibody levels in the valley were twice as high as that observed in the uphill population (Mann Whitney; p < 0.001; Figure 2A). Furthermore seasonal variation was also observed in antibody levels (Mann Whitney; p = 0.028; Figure 2B), with higher levels during rainy season.

The risk of seroconversion to MSP-1_19_ specific Ab, following exposure to *Plasmodium falciparum* was always higher for gSG6-P1 seropositives than seronegative individuals (Table 1). Altogether the odds of gSG6-P1 seropositives having MSP-1_19_ Ab were almost three times higher than the odds of seronegatives. This was significant in the uphill population by a factor of 2.2 and highly so in the valley population by a factor of 2.6 (Table 1).

**Table 1:** Association between gSG6-P1 and MSP-1_19_ seroprevalence at the different localities

	**MSP-1**_**19**_**seroprevalence**		
**gSG6-P1 seroprevalence**			
***Locality (n)***	***Odds Ratio***	***95% CI***	***P value***
Uphill (232)	2.168	[1.203, 3.903]	0.010
Valley (375)	2.668	[1.550, 4.592]	< 0.001
Total Uphill and Valley (607)	2.873	[1.977, 4.176]	< 0.001

### Age trends in parasite prevalence, specific antibody responses to gSG6-P1, and MSP-1_19_

**Figure 3:**
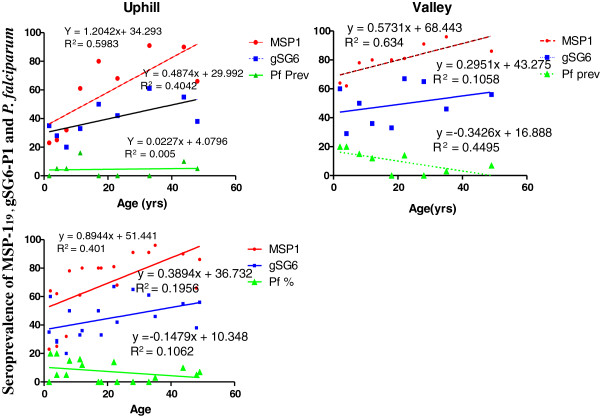
**A scatter plot showing the trends of gSG6-P1 and MSP-1**_**19**_**seroprevalence together with P*. falciparum *with age at different locations.** A (Uphill n = 232), B (valley bottom n = 375) and C (Total population n = 607).

There was no correlation observed between parasite prevalence and age in the uphill population (Figure 3A), however there was a strong negative correlation between the parasite prevalence and age in the valley population (Figure 3B), and when the data was combined (Figure 3C). Parasite prevalence was generally very low relative to seroprevalence of gSG6- P1 and MSP-1_19_ at all study sites (Figure 3)_._ Linear regression analysis revealed significant differences in the slopes in the uphill population (F = 5.2, df = 2, 21, P = 0.014), Valley population (F = 4.3, df = 2, 21, P = 0.026) and when both populations were analysed together (F = 6.5, df = 2, 48, P = 0.003). MSP-1_19_ seroprevalence was strongly associated with age at individual sites and in total (Figure 3). The correlation between gSG6-P1 specific seroprevalence and age was considerably high in the uphill population (R^2^ = 0.40, Figure 3A), the contrast was observed in the valley bottom residents particularly so in the valley population (R^2^ = 0.10, Figure 3B) and again when the data was combined (R^2^ = 0.19, Figure 3C).

The differences in the magnitude of responses between MSP -1_19_ and gSG6-P1 specific antibodies, as indicated by the differences in their intercept are highly significant at both sites and when the data was combined (Figure 3). However, antibody trends as depicted by the slopes did not differ.

### Specific antibody responses to gSG6-P1 across different transmission settings and risk of parasite exposure

**Figure 4:**
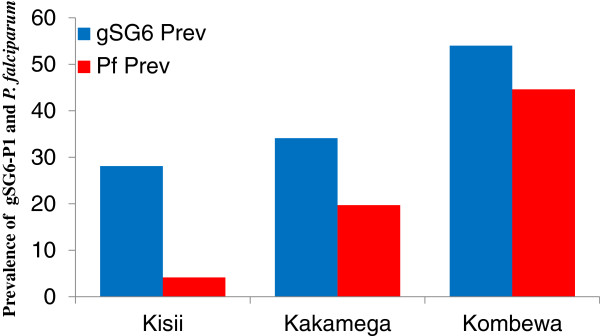
**A bar graph showing gSG6-P1 seroprevalence and their respective parasite prevalence in different endemic localities.** Kisii (n = 222), Kakamega (n = 203) and Kombewa (n = 202).

We observed significant differences in parasite prevalence and gSG6-P1 levels across different transmission settings (Figure 4). The hypoendemic Kisii area had the lowest parasite prevalence of barely 4% followed by the mesoendemic area of Kakamega with 19.7%. Parasite prevalence was highest in the hyperendemic Kombewa area at 44.6% (χ^2^ = 31.0 df = 2, P < 0.001). The observed parasite prevalence was associated with increasing gSG6-P1specific antibody prevalence along the transmission intensity cline of Kisii (28%), Kakamega (34%) and Kombewa (54%) (χ^2^ = 99.0 df = 2, P < 0.001). The two measurements were significantly positively associated (χ^2^ = 10.9, df = 2, P = 0.004).

## Discussion

Marked differences in gSG6-P1 specific seroprevalence and antibody levels were observed between the uphill and valley bottom populations. The IgG levels of to gSG6-P1 in the valley bottom population were two-fold higher than observed in the uphill population, suggesting that, the valley bottom population is exposed to higher vector densities than the uphill population. Seasonally, antibody levels of gSG6 P1 in the rainy season were higher than the levels observed in the dry season; however this was not the case in the seroprevalence. It has been previously shown that the levels of specific IgG response to this peptide reflect recent exposure; antibody levels tend to be higher in actively exposed individuals with a concomitant decline in the absence of exposure [40]. The differences in the seroprevalence and Ab levels to gSG6-P1 may be attributed to the differences in vector densities between the two sites; majority of breeding habitats in the hilly highlands are confined to the valley bottoms because the hillside gradients provide efficient drainage [41]. Githeko *et al.*[33] found that, the majority of the adult *Anopheles* females *An. gambiae s.l*. 98% and *An. funestus* 99%], were found up to 500 m from the breeding habitats which were clustered in the valley bottom. Very few vectors are found at mid hill (1%) and at the hill top (1%). It is well known that the topography of the highlands in western Kenya play a major role in malaria prevalence as this difference between the two sites has been reported by other studies [33,34]***.*** In respect to this evidence it may be indicated that the gSG6-P1 biomarker is robust and sensitive enough to distinguish between the vector densities of exposure in a population which is only 5 km apart.

Regression analysis revealed that, the odds of having detectable anti-MSP-1_19_ Ab response was significantly higher for gSG6-P1 seropositive individuals. This implies that, the risk of exposure to malaria parasite is higher in individuals presenting with anti -gSG6 P1 Ab; this was consistent both in the uphill and valley residents. With the known differences in vector exposure between the two sites, it is conservable to say that, higher exposure to vector results in higher risk of parasite transmission. This conclusion is consistent with an established view of malaria epidemiology; that the risk of receiving a mosquito bite as well as susceptibility to infection are highly heterogeneous and that 20% of people receive 80% of all bites and infections [42,43].

Indeed it was exactly the case when we compared anti -gSG6-P1 IgG responses in school children from three different malaria transmission intensity zones. Marani, Kakamega and Kombewa, where their respective EIR are as follows: 0.4, 16.6 and 31.1 [35]. In the same order we observed parasite rates of 4%, 19.7% and 44.6%. The observed gSG6-P1 seroprevalence significantly declined with level of endemicity (Kombewa (54%), Kakamega (34.1%) and Marani (28.1%) which paralleled the parasites rates (Figure 4). However the gSG6-P1 seroprevalence rates relatively higher being indicative of higher sensitivity.

At the population level, we have observed that gSG6-P1 seroprevalence may correlate with the risk of pathogen transmission, in agreement with previous results showing that high anti-saliva IgG levels were predictive indicators of malaria morbidity [26]. Anti-*Anopheles dirus* salivary proteins Ab occurred also predominantly in patients with acute *P. falciparum* or *Plasmodium vivax* malaria compared to individuals from non-endemic areas [27]. In South Americas, the presence of anti-*Anopheles* saliva Ab has been described in malaria-endemic areas. Adult volunteers from communities in the state of Rondônia, Brazil, were tested for Ab response against *Anopheles darlingi* salivary gland sonicates (SGS). Individuals infected with *P. vivax* presented higher levels of anti-SGS Ab than did non-infected individuals. This potential biomarker appeared thus useful as an epidemiological tool for discriminating between infected and non-infected individuals with a high likelihood ratio [44].

As expected, MSP-1_19_ seroprevalence was strongly associated with age at individual sites and in general. The longevity and the cumulative nature of anti-MSP-119 Ab responses is well known. However the age trends observed for gSG6-P1 responses in the same cohort was intriguing. The overall correlation between anti- gSG6-P1 Ab seroprevalence and age was very weak, particularly so in the valley bottom population, however these trends were considerable in the uphill population (Figure 3). The lack of correlation in the age trends of gSG6-P1seroprevalence in the overall data as well as the valley bottom residents in comparison to MSP-1_19_ may be informative that anti - gSG6-P1 Ab is not cumulative. It is noteworthy that age trends of gSG6-P1is influenced by the differences in the transmission intensity. In hyperendemic areas of Burkina Faso, Rizzo and others found that children had higher responses to whole salivary gSG6 proteins while adults had diminished Ab responses, suggesting desensitization of the immune response to the salivary proteins [29]. The current study confirms the non cumulative nature of Ab response gSG6-P1 peptide and thus its robustness in measuring transient exposure (or seasonal) in a hypoendemic population as observed in our uphill residents and not only restricted to children in hyperendemic areas. It will add to the advantages of gSG6-P1 as it will be more useful even under low malaria transmission period as envisaged in the pre elimination and elimination phase of malaria.

MSP-1_19_ Ab responses appeared largely higher than anti- gSG6-P1 responses in terms of Ab level. The differences further lends credence to the observation that MSP1 is cumulative or perhaps more immunogenic than gSG6-P1. Furthermore, the amount of gSG6 proteins injected in the blood as well as the time of contact with immuno-competent cells is relatively shorter than MSP1_19_ which is a blood stage antigen and multiples severally during the erythrocytic cycle of the parasite.

The gSG6-P1 is a synthetic peptide specially designed to enhance its sensitivity and immunogenicity [21], and it has reportedly been very sensitive [25,28] and highly immunogenic developing immune responses even in travelers only transiently exposed to mosquito bites.

## Conclusion

gSG6 P1 seroprevalence correlates with parasite prevalence at the population level. The seroprevalence of gSG6-P1 was sensitive and robust to distinguish between hypo, meso and hyper transmission settings and the level of specific Ab distinguished between seasonal fluctuations. The gSG6 P1 seroprevalence may be exploited as an epidemiological marker of risk of parasite transmission and a vector surveillance tool across different populations and malaria transmission settings.

## Competing interests

The authors declare that they have no competing interests.

## Authors’ contributions

KB carried out the field survey, the serological experiments, and the serological analysis and wrote the first draft of the manuscript. JS and FR supervised laboratory procedures and assay development; GZ supported data analysis. KB, GY, BWL and, AG conceived the study, participated in its design and implementation and JL, FR and JMO contributed to data analysis and manuscript development. YA coordinated the collection of samples. All authors read and approved the final manuscript.
